# Global metabolomic and lipidomic analysis reveals the potential mechanisms of hemolysis effect of Ophiopogonin D and Ophiopogonin D' in vivo

**DOI:** 10.1186/s13020-020-00412-z

**Published:** 2021-01-06

**Authors:** Huan-Hua Xu, Zhen-Hong Jiang, Cong-Shu Huang, Yu-Ting Sun, Long-Long Xu, Xiang-Ling Tang, Hong-Ling Tan, Zeng-Chun Ma, Yue Gao

**Affiliations:** 1 Tianjin University of Traditional Chinese Medicine, Tianjin, 300193 China; 2 Department of Pharmaceutical Sciences, Beijing Institute of Radiation Medicine, Beijing, 100850 China; 3 Jiangxi Province Key Laboratory of Molecular Medicine, Nanchang, 330006 China; 4 Guangdong Pharmaceutical University, Guangzhou, 510006 China; 5 College of Life Science and Bioengineering, Beijing University of Technology, Beijing, 100124 China

**Keywords:** Hemolysis, Ophiopogonin D, Ophiopogonin D', Metabolomic, Lipidomic

## Abstract

**Background:**

OPD and OPD' are the two main active components of Ophiopogon japonicas in Shenmai injection (SMI). Being isomers of each other, they are supposed to have similar pharmacological activities, but the actual situation is complicated. The difference of hemolytic behavior between OPD and OPD' in vivo and in vitro was discovered and reported by our group for the first time. In vitro, only OPD' showed hemolysis reaction, while in vivo, both OPD and OPD' caused hemolysis. In vitro, the primary cause of hemolysis has been confirmed to be related to the difference between physical and chemical properties of OPD and OPD'. In vivo, although there is a possible explanation for this phenomenon, the one is that OPD is bio-transformed into OPD' or its analogues in vivo, the other one is that both OPD and OPD' were metabolized into more activated forms for hemolysis. However, the mechanism of hemolysis in vivo is still unclear, especially the existing literature are still difficult to explain why OPD shows the inconsistent hemolysis behavior in vivo and in vitro. Therefore, the study of hemolysis of OPD and OPD' in vivo is of great practical significance in response to the increase of adverse events of SMI.

**Methods:**

Aiming at the hemolysis in vivo, this manuscript adopted untargeted metabolomics and lipidomics technology to preliminarily explore the changes of plasma metabolites and lipids of OPD- and OPD'-treated rats. Metabolomics and lipidomics analyses were performed on ultra-high performance liquid chromatography (UPLC) system tandem with different mass spectrometers (MS) and different columns respectively. Multivariate statistical approaches such as principal component analysis (PCA) and orthogonal partial least square-discriminant analysis (OPLS-DA) were applied to screen the differential metabolites and lipids.

**Results:**

Both OPD and OPD' groups experienced hemolysis, Changes in endogenous differential metabolites and differential lipids, enrichment of differential metabolic pathways, and correlation analysis of differential metabolites and lipids all indicated that the causes of hemolysis by OPD and OPD' were closely related to the interference of phospholipid metabolism.

**Conclusions:**

This study provided a comprehensive description of metabolomics and lipidomics changes between OPD- and OPD'-treated rats, it would add to the knowledge base of the field, which also provided scientific guidance for the subsequent mechanism research. However, the underlying mechanism require further research.

## Background

Shenmai injection (SMI) is a widely used traditional Chinese medicine (TCM) extracted from Panax ginseng and Ophiopogon japonicas [1]. It has been widely used to treat different disease for its excellent therapeutic effects, such as the shock of qi and yin deficiency [2, 3], coronary heart disease [4, 5], viral myocarditis [6], chronic pulmonary heart disease [7], and neutropenia [8]. It can also strengthen the immune system of cancer patients to help prevent cancer [9, 10]. When used in combination with chemotherapy drugs, it has a certain synergistic effect and can reduce the side effects caused by chemotherapy drugs [11]. In the pneumonia pandemic caused by the new coronavirus (COVID-19) in early 2020 in China [12], SMI was listed as a recommended medication due to its positive effect on the improvement of patients’ blood oxygen saturation by the Novel Coronavirus Pneumonia Diagnosis and Treatment Plan (Provisional 7th Edition), which was released by the Chinese government [13].

**Fig. 1:**
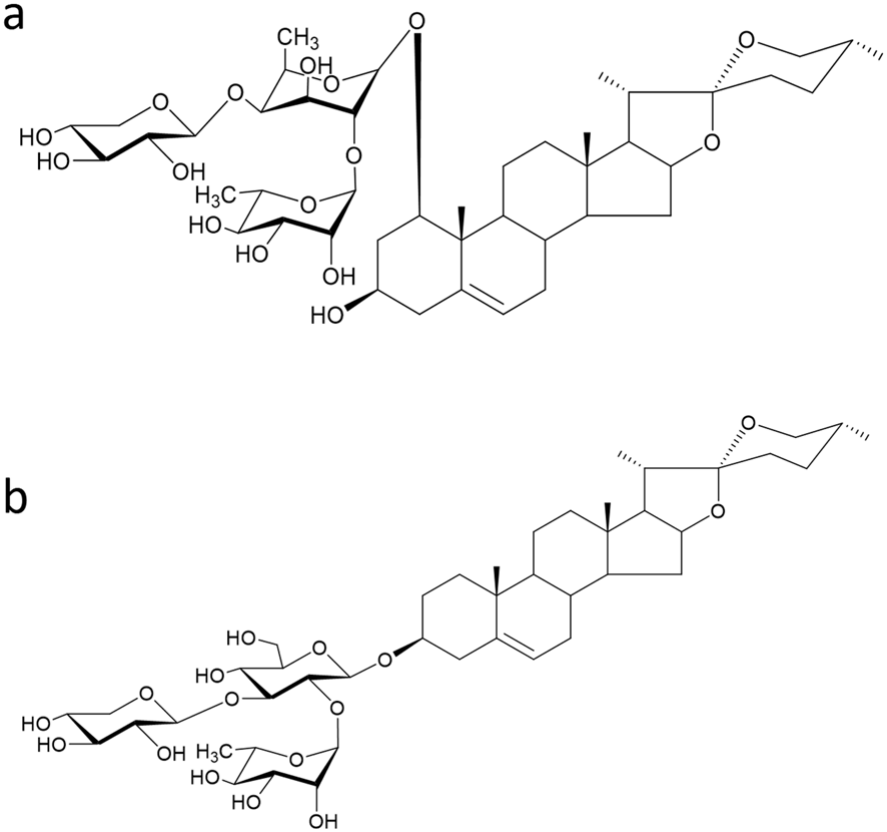
The chemical structure of Ophiopogonin D (OPD) and Ophiopogonin D' (OPD'). **a** Ophiopogonin D: Ruscogenin 3-O-{α-L-rhamnopyranosyl(1 → 2)-[β-D-xylopyranosyl (1 → 3)]-β-D-fucopyranoside}. **b** Ophiopogonin D′: Diosgenin 3-O-{α-L-rhamnopyranosyl(1 → 2)-[β-D-xylopyranosyl (1 → 3)]-β-D-glucopyranoside}. Chemical formula: C44H70O16. MW = 854.

Ophiopogonin D (OPD) and Ophiopogonin D' (OPD') are two main active components in Ophiopogon japonicas with the same molecular formula and similar structure, only show the different substitution of sugar side chains (Fig. 1). Previous studies on active components of Ophiopogon japonicas always focused on OPD, and have found that OPD was an effective ingredient for the treatment of cardiovascular and cerebrovascular diseases [14, 15]. OPD has antioxidant activity and can promote cell proliferation, stabilize mitochondrial membrane potential, reduce calcium influx, inhibit angiotensin II as well as induce liver microsomal enzymes [16]. However, since 2017, there have been reports of cytotoxicity of OPD' at the cellular level. Lu's et.al found that OPD' could significantly inhibit the growth of androgen-independent prostate cancer PC3 cells, and was related to the RIP1/MLKL pathway [17, 18]. When H9c2 [19] cardiomyocytes and HepG2 [20] human hepatoma cells were treated with OPD' (> 5 μmol·L^−1^) for 24 h, cells were suffered from cell morphology shrank, the number of nuclei decreased, ROS content increased, mitochondrial membrane potential decreased and the rate of apoptosis increased. The survival rate of cells gradually decreased with the extension of the treatment time of OPD', and the toxic reaction became more and more obvious.

In our previous work, we discovered for the first time that the hemolytic behavior of OPD' is completely different from OPD (unpublished manuscript). Despite SMI was widely used in clinical and has gained a good reputation, the risk assessment of SMI based on cytotoxicity and hemolytic of OPD' is still missing, limiting our ability to improve the safety of SMI. Also, with the widespread clinical application, the adverse reaction events of SMI are increasing. From January 2012 to December 2014, only the Hubei Provincial Adverse Drug Reaction Testing Center collected more than 1,500 cases of adverse reactions reports of SMI [21]. Existing adverse event analysis for the collected SMI indicates that there was a great correlation between adverse events and hemolysis. But the mechanism of hemolysis in vivo is still vague.

Here, we further specific manifestations in hemolytic behavior between OPD and OPD' in vivo, and untargeted metabolomics and lipidomics were adopted to study the underlying mechanisms. In the in vivo hemolysis test, both OPD and OPD' affected the content of hemoglobin (HGB), red blood cell (RBC) count and reticulocyte (RET) count in the blood. We also first identified different metabolites by pairwise comparison of different groups, and then further classified the differential metabolites by function. We found that both OPD and OPD' could induce hemolysis in vivo by interfering with phospholipid metabolism, promoting the production of lysophospholipids (LysoPC), and causing damage to the red blood cell membrane. Although the direct cause of hemolysis is inseparable from the disorder of phospholipid metabolism, however, how OPD and OPD' participated in this process needs further exploration.

## Materials and methods

### Chemicals and reagents

OPD (F05975, purity: 98% by HPLC) and OPD' (F581298, purity: 98% by HPLC) were purchased from Shanghai EFE Biotechnology Co., Ltd. (Shanghai, China). Qualitative detection kit for urine hemosiderin test (Rous) was purchased from Shanghai Yuan Mu Biotechnology Co., Ltd. (Shanghai, China).

All other chemicals and solvents were analytical or HPLC grade. The ultra-pure water was prepared by the Milli-Q system (Millipore, Bedford, MA, USA). Methanol, acetonitrile, formic acid were purchased from CNW Technologies. GmbH (Düsseldorf, Germany). L-2-chlorophenylalanine was purchased from Shanghai Heng Chuang Bio-technology Co., Ltd. (Shanghai, China).

### Animals

In vivo hemolytic study was conducted in the apparently healthy adult Wistar rats (180-200 g, male and female). Animals were purchased from Beijing Charles River Animal Breeding Center (Production license: SCXK (Jing)-2018-0010). All animals were housed in an environmentally controlled breeding room (temperature: 22 ± 2 ℃, humidity: 50 ± 5%, dark/light cycle: 12/12 h). The animals were provided standard laboratory food and water. The experimental protocols were approved by the Animal Ethics Committee of Academy of Military Medical Sciences (No. IACUC-DWZX-2020-684) and were performed in accordance with the guidelines of the National Institutes of Health for the Care and Use of Laboratory Animals.

### Parameters related to hemolysis

In this experiment, the groups were set as follows: normal control group (NC), OPD independent use group (OPD), OPD' independent use group (OPD'), OPD and OPD' combination group (OPD + OPD'), OPD and OPD' interval administration group (OPD → OPD'). 25 Wistar rats (5 rats in each group) were given by tail vein injection once a day for 30 days. The activities and physiological conditions of rats were observed and recorded every day, body weight were weighed every 7 days. At the end of experiments, carbon dioxide anesthesia, whole blood was collected, anticoagulation and perform hematology analysis. Plasma was separated by centrifugation for biochemical analysis. Urine were collected, one part was used to determine whether it contains hemosiderin by Rous method, another part was used for routine urine testing.

### Metabolomic experiments

#### Sample preparation

22 rats were randomly assigned to 3 groups, they are normal control group (NC, n = 6), OPD group (n = 8) and OPD' group (n = 8). All drugs were administrated by intravascular injection for 14 days. According to the limit of clinical hemolysis rate (5%) and the results obtained from in vitro hemolytic test, When the concentration of OPD' exceeds 5 μg·ml^−1^, the hemolysis rate exceeds the clinical limit of 5%. Assuming an ideal situation, according to the blood volume of the experimental animals and this blood drug concentration (i.e. 5 μg·ml^−1^), the doses of the OPD and OPD' of each group are defined as 0.25 mg·kg^−1^. Plasma was prepared by centrifugation for metabolomics analysis.

100μL of plasma was added to a 1.5 mL Eppendorf tube with 10 μL of 2-chloro-1-phenylalanine (0.3 mg·mL^−1^) dissolved in methanol as internal standard. Subsequently, 300 μL of ice-cold mixture of methanol and acetonitrile (2/1, v/v) was added, and the mixtures were vortexed for 1 min, ultrasonicated at ambient temperature (25–28 ℃) for 10 min, store at − 20 ℃ for 30 min. The extract was centrifuged at 13,000 rpm, 4 ℃ for 15 min, 300 μL of supernatant in a brown and glass vial was dried in a freeze concentration centrifugal dryer, 400 μL mixture of methanol and water (1/4, v/v) were added to each sample and then vortexed for 30 s, Repeat the centrifugation operation, 150 μL supernatants from each tube were collected using crystal syringes, filtered through 0.22 μm microfilters and transferred to LC vials for LC–MS analysis. QC samples were prepared by mixing aliquots of the all to be a pooled sample and the QCs were injected at regular intervals (every 10 samples) throughout the analytical run to provide a set of data from which repeatability can be assessed.

#### UPLC-MS/MS analysis

Instrument for this study is a LC–MS system composed of Dionex U3000 UPLC ultra-efficient liquid chromatography tandem a QE plus high-resolution mass spectrometer. Liquid phase separation was performed on an Acquity UPLC HSS T3 (100 mm × 2.1 mm, 1.8 µm) column. The flow rate was controlled at 0.35 mL·min^−1^, the temperature of the automatic injector was maintained at 4 ℃, and the column temperature was controlled at 50 ℃. The injection volume of all samples was 5 μL. The mobile phase consisted of water and acetonitrile which both containing 0.1% formic acid. Gradient elution was performed as follows: (1) mobile phase A was at 95% at 0 min, (2) an isocratic elution was maintained at 95% A from 0 to 1 min, (3) a linear gradient was decreased to 0% A from 1 to 11 min, and (4) mobile phase A maintained at 0% from 11 to 13 min.

The condition of mass spectrometry (MS) analysis was ESI source ionization in positive and negative ion mode respectively, and the scanning mode adopted centroid and continuum mode. The optimum conditions were shown in Table 1.

**Table 1:** The condition of mass spectrometry (MS) analysis

Parameter	ESI positive	ESI negative
Spray Voltage (V)	3800	3000
Capillary Temperature (°C)	320	320
Aux gas heater temperature (℃)	350	350
Sheath Gas Flow Rate (Arb)	35	35
Aux gas flow rate (Arb)	8	8
S-lens RF level	50	50
Mass range (m/z)	70–1000	70–1000
Full ms resolution	70,000	70,000
MS/MS resolution	17,500	17,500
NCE/stepped NCE	20, 40	20, 40

### Lipidomic experiments

#### Sample preparation

After the same steps of metabolomic sample preparation. 100 μL of plasma was added to a tube with 20 μL of 2-chloro-1-phenylalanine (0.3 mg·mL^−1^) and Lyso PC (17:0) (0.01 mg·mL^−1^) dissolved in methanol as internal standard. Subsequently, 300 μL Isopropanol was added, and vortex for 30 s, and extract with ultrasound for 10 min. Place at – 20 ℃ for 30 min, centrifuge for 10 min, take 300 μL of supernatant and transfer to a new tube. Another 200 μL of isopropanol was added to the original tube and extract again, take 200 μL up layer and merge it into the new tube. After drying, the lipid residue in the centrifuge tube was reconstituted with 200μL isopropanol-methanol mixture (1:1, V/V), vortex 30 s, ultrasound 3 min, centrifuged for 10 min (12000 rpm, 4 ℃), take 150 μL of the supernatant and put it into an LC–MS sample vial for LC–MS analysis. QC samples were prepared by mixing equal volumes of extracts from all samples, and each QC has the same volume as the sample.

#### UPLC-MS/MS analysis

The lipidomic analyses were performed on Q Exactive Mass Spectrometer (Thermo Fisher Scientific Inc., Waltham, MA, USA) coupled to Nexera UPLC system (Shimadzu, Kyoto, Japan). An ACQUITY UPLC BEH C18 (2.1 mm × 100 mm, 1.7 μm, Waters, Milford, MA, USA) was used for separation of lipids. The mobile phase for HPLC was composed of solvent A (10 mM ammonium formate and 0.1% formic acid in acetonitrile/water (60:40)) and solvent B (10 mM ammonium formate and 0.1% formic acid in isopropanol/ acetonitrile (90:10)). LC gradient was as follows: 0.0–3.0 min for 30% B, 3.0–5.0 min to 62% B, 5.0–15.0 min to 82% B, 15.0–16.5 min to 99% B, 16.5–18.0 min to maintain 99% B. The flow rate was 0.35 mL/min, and the injected sample amount was 5μL. Heated electrospray ionization (HESI) positive and negative ion modes were used for detection. Positive: Heater Temp 300 °C, Sheath Gas Flow rate 45 arb, Aux Gas Flow Rate15 arb, Sweep Gas Flow Rate 1arb, spray voltage 3.5KV, Capillary Temp 320 °C, S-Lens RF Level 50%. MS1 scan ranges: 120–1800. Negative: Heater Temp 300 °C, Sheath Gas Flow rate 45arb, Aux Gas Flow Rate 15arb, Sweep Gas Flow Rate 1arb, spray voltage 3.1KV, Capillary Temp 320 °C, S-Lens RF Level 50%. MS1 scan ranges: 120–1800.

### Data processing and statistical analysis

The acquired LC–MS raw data were analyzed by the progqenesis QI software (Waters Corporation, Milford, USA). The Excel file was obtained with three dimensions datasets including gm/z, peak RT and peak intensities, and RT–m/z pairs were used as the identifier for each ion. The resulting matrix was further reduced by removing any peaks with missing value (ion intensity = 0) in more than 50% samples.

Metabolites were identified by progenesis QI (Waters Corporation, Milford, USA) Data Processing Software, based on public databases such as http://www.hmdb.ca/; http://www.lipidmaps.org/ and self-built databases. The software Lipid search (Thermo Fisher Scientific Inc., Waltham, MA, USA) was employed for peak picking and alignment of lipids.

The positive and negative data were combined to get a combine data which was imported into R package. Principle component analysis (PCA) and (orthogonal) partial least-squares-discriminant analysis (OPLS-DA) were carried out to visualize the metabolic alterations among experimental groups, after mean centering and Pareto variance scaling, respectively. The Hotelling’s T2 region, shown as an ellipse in score plots of the models, defines the 95% confidence interval of the modeled variation. Variable importance in the projection (VIP) ranks the overall contribution of each variable to the OPLS-DA model, and those variables with VIP > 1 are considered relevant for group discrimination.

## Results

### The hemolytic behavior of OPD and OPD' in vivo

**Fig. 2:**
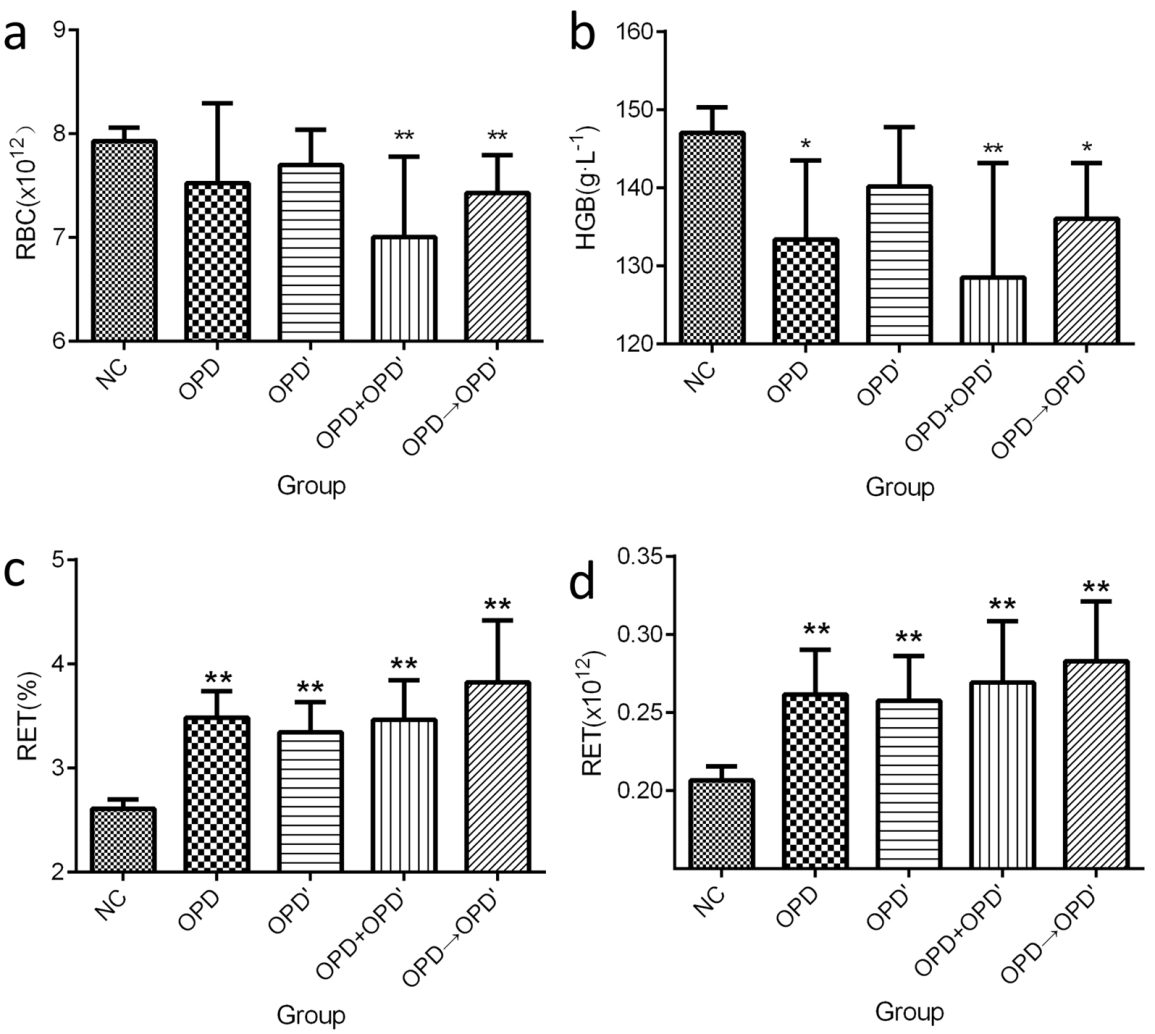
Parameters related to hemolysis in each group by hematology analysis. **a** RBC count. **b** HGB content. **c** RET count. **d** RET%. The error bars represent the standard deviation of measurements for 5 parallel samples in five separate groups (n = 5). “*” means that compared with the NC group, the statistical was significant (*p* < 0.05), “**” means that compared with the NC group, the statistical was very significant (*p* < 0.01)

As it was shown in Fig. 2 and Table 2, Hemolysis was found in each drug treatment group, and hematological indicators related to hemolysis, such as RBC, HGB, RET, and RET%, were significantly changed. Compared with the NC group, RBC and HGB decreased significantly, while RET and RET% were significantly increased, which means that RBCs were destroyed faster, and the immature RBCs (i.e. RET) were released to the peripheral blood increased. Indicated that the lifespan of RBC may be shortened. The results of the urine examination showed that the urobilinogen (URO) of each treatment group changed significantly, and compared with the NC group, they were all positive (degree 1 + to 2 + . Under normal circumstances, there should be no red blood cells, urobilinogen and hemosiderin in the urine, which is represented by “-”. However, with the occurrence and aggravation of hemolysis, the number and concentration of RBC, URO, and hemosiderin will increase, expressed on a scale from 1 + to 4 + .). The results of the urine occult blood tests were consistent with the results of the URO test. The Rous method [22] was used to determine the hemosiderin in urine, which is a commonly used clinical test to diagnose intravascular hemolysis. As shown in Table 2, obviously scattered or piles of blue glitter particles were observed in each treatment group (degree 1 + to 2 +). These indicators confirm that OPD and OPD' can cause intravascular hemolysis [23] when used alone or in combination.

**Table 2:** Parameters related to hemolysis in each group by urine examination

Group	Hemosiderin	RBCs in urine	Urobilinogen
NC	−	−	−
OPD	+	+	+
OPD'	+ +	+	+ +
OPD + OPD'	+ +	+ +	+ +
OPD → OPD'	+ +	+ +	+ +

### Global metabolic and lipidomic shifts induced by OPD and OPD'

**Fig. 3:**
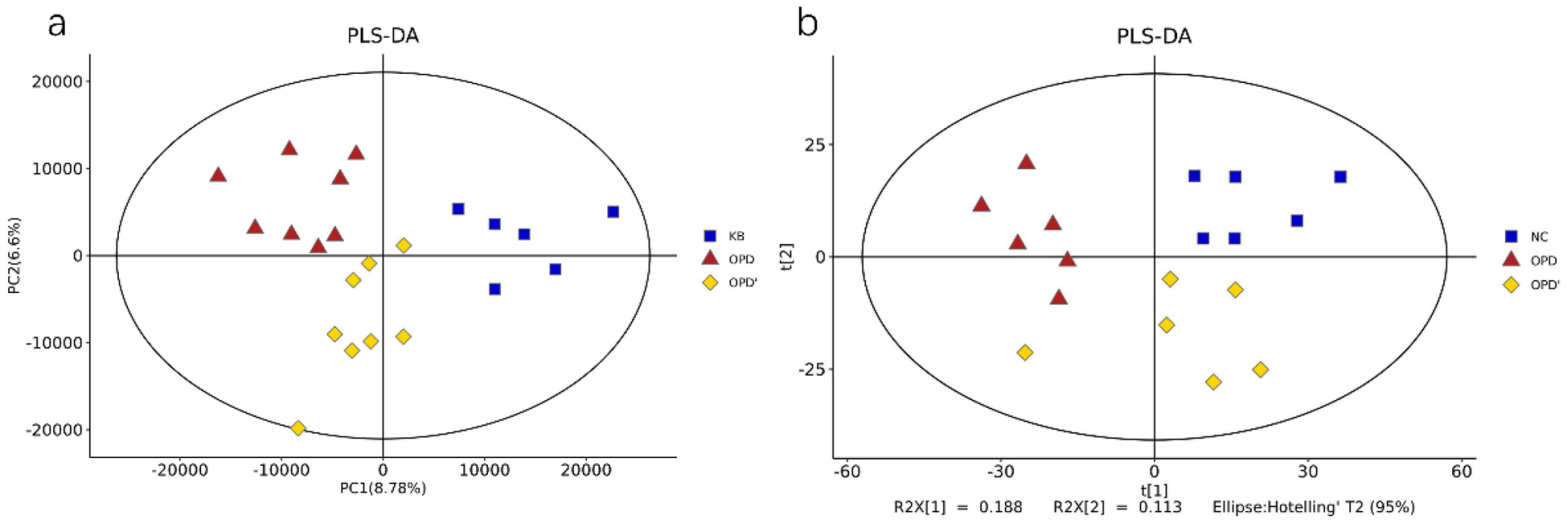
Two-dimensional PLS-DA score plots of metabolic and lipidomic profiles in vivo with different drug treatment. **a** Metabolomic analysis in ESI positive and negative mode. **b** Lipidomic analysis in ESI positive and negative mode

The metabolic and lipidomic profiles were acquired under positive and negative ionization modes using UPLC-MS. A total of 10,364 ions in ESI ( +) and 9741 ions in ESI (-) were obtained by non-targeted metabolic profiling. In lipidomic profiling, 313 ions in ESI ( +) and 260 ions in ESI (−) were obtained. PLS-DA score plots were applied to identify the differential metabolites or lipids among OPD and OPD' groups. Figure 3 showed the clear segregation of OPD and OPD' from NC group, indicating that both OPD and OPD' induced obvious disturbance of inter-cellular metabolites and lipids.

### Metabolic changes induced by OPD and OPD' in vivo

**Fig. 4:**
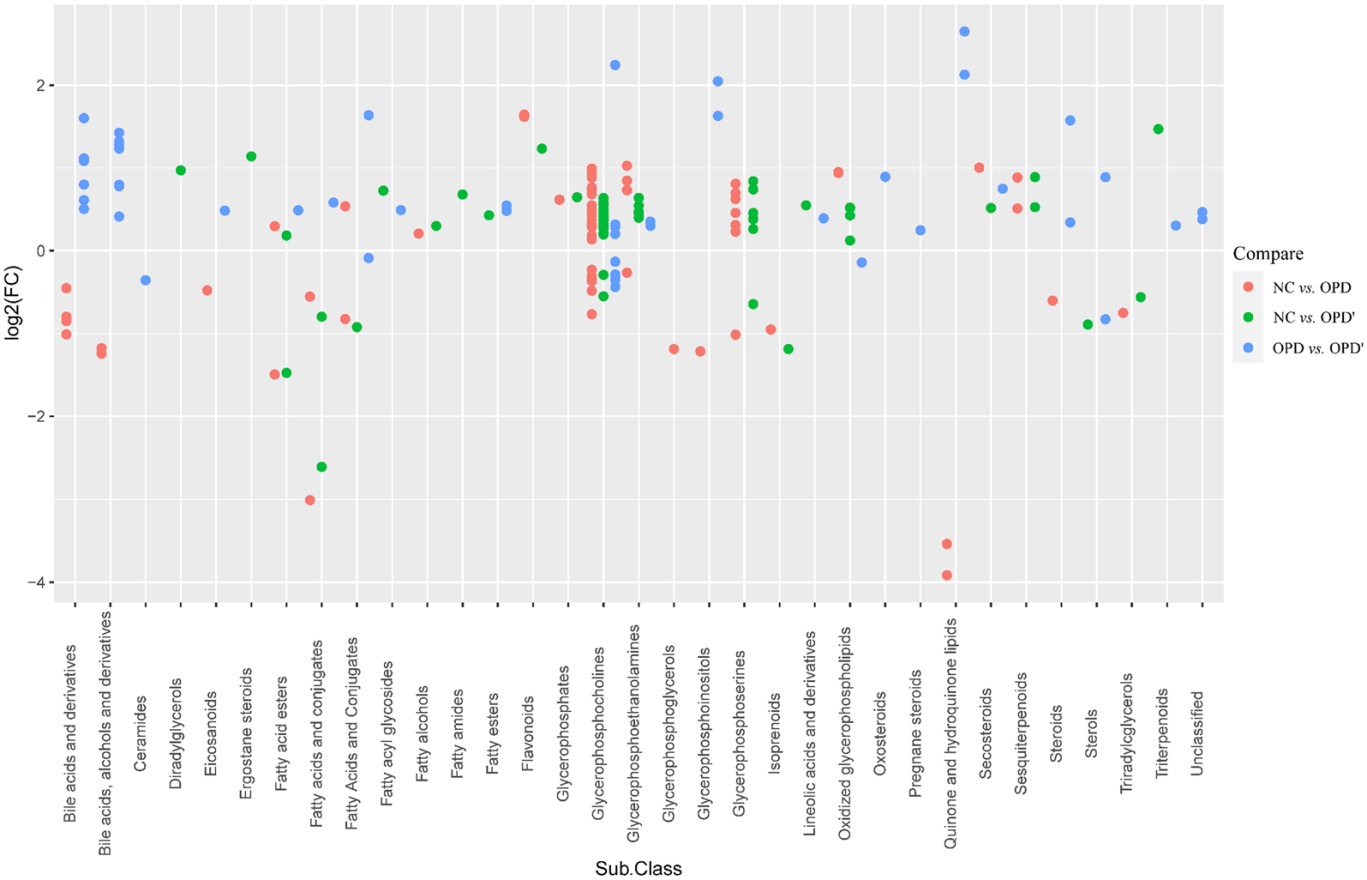
The fold changes of differential metabolites among each comparison group from the perspective of sub class

According to OPLS-DA model, Variable importance of projection (VIP) value was obtained, and VIP > 1 is the one of criterion for screening potential biomarkers. In each comparison group, the number of differential metabolites we found were 145 (NC vs OPD), 124 (NC vs OPD') and 84 (OPD vs OPD'), respectively. The volcano graph can be used to visualize the p value and Fold change value, which is helpful for screening different metabolites (Additional file 1: Figures S1 and S2). Top 20 differential metabolites were listed in Table 3 among different comparison group, and the distribution of fold changes of metabolites in different comparison group from the perspective of sub-class were shown in Fig. 4.

**Table 3:** Detailed information of top 20^a^ perturbed metabolites among different comparison group

Metabolites	m/z	Retention time (min)	Detection mode	(NC vs OPD)		(NC vs OPD')		(OPD vs OPD')	
				VIP	FC	VIP	FC	VIP	FC
LysoPC (18:1(9Z))^b^	522.3554	10.4072	Positive	40.29	1.23	42.27	1.22	/	/
PC(16:0/0:0)[U] / PC(16:0/0:0)^b^	496.3396	9.8993	Positive	/	/	22.25	1.13	/	/
Cholic acid^b^	815.5688	8.2063	Negative	/	/	/	/	28.13	1.72
(R)-Butaprost	834.6078	8.2002	Positive	/	/	/	/	20.9	1.55
PC (14:0/0:0)^b^	468.3084	9.0744	Positive	17.68	1.95	13.98	1.48	6.50	0.76
LysoPC (16:1(9Z)/0:0)^b^	538.3154	9.3950	Negative	14.43	1.92	11.36	1.41	7.53	0.74
LysoPC (20:2(11Z,14Z))^b^	548.3709	10.6952	Positive	14.12	1.30	16.65	1.34	/	/
LysoPC (20:1(11Z))^b^	550.3865	11.3843	Positive	12.81	1.22	16.56	1.32	/	/
1-heptadecanoyl-sn-glycero-3-phosphocholine^b^	510.3555	10.6233	Positive	/	/	15.9	1.15	/	/
LysoPC(16:0)^b^	540.3312	9.8953	Negative	/	/	11.4	1.14	/	/
3alpha-Hydroxy-5beta-chola-7,9(11)-dien-24-oic Acid	373.2735	8.2002	Positive	12.72	0.73	/	/	20.47	1.53
LysoPC (22:5(7Z,10Z,13Z,16Z,19Z))^b^	570.3551	10.0146	Positive	12.70	1.36	8.09	1.29	/	/
15-hydroxy-tetracosa-6,9,12,16,18-pentaenoic acid	357.2785	9.3971	Positive	11.09	0.56	/	/	/	/
Sulfolithocholylglycine	512.2690	6.0969	Negative	/	/	/	/	9.80	1.74
LysoPC (20:3(5Z,8Z,11Z))^b^	590.3467	10.1463	Negative	10.17	1.46	8.86	1.3	/	/
PC (17:1(10Z)/0:0)^b^	508.3393	9.8993	Positive	9.18	1.67	7.65	1.37	3.54	0.82
(22E)-3beta-Hydroxy-5alpha-chola-7,22-dien-24-oic Acid	373.2735	7.4742	Positive	9.18	0.57	/	/	14.6	2.12
LysoPC (14:0/0:0)^b^	512.2997	9.0829	Negative	8.99	1.99	6.92	1.47	4.15	0.74
PC (19:1(9Z)/0:0)^b^	536.3708	10.8693	Positive	8.90	1.61	8.55	1.51	/	/
Isocitrate	191.0189	0.7660	Negative	8.35	1.74	/	/	/	/
PC(O-18:1(11Z)/0:0)^b^	508.3762	10.7094	Positive	8.22	0.79	/	/	/	/
LysoPC (17:0)^b^	554.3466	10.6250	Negative	7.90	1.14	10.33	1.21	/	/
3alpha,7alpha,12alpha-Trihydroxy-5alpha-cholan-24-al	783.5790	9.3950	Negative	7.50	0.50	/	/	/	/
2-Hydroxycinnamic acid	182.0810	1.1162	Positive	6.38	1.16	8.68	1.22	/	/
Avicholic acid	391.2840	7.6371	Positive	5.47	0.58	/	/	3.91	1.42
LysoPC (20:5(5Z,8Z,11Z,14Z,17Z))^b^	586.3156	9.2737	Negative	4.98	1.41	/	/	4.66	0.78
3alpha,9alpha,11beta-Trihydroxy-5beta-cholan-24-oic Acid	391.2840	7.4742	Positive	/	/	/	/	5.62	2.17
PC(16:0/18:2(9Z,12Z))^b^	780.5518	13.6416	Positive	/	/	6.42	0.68	/	/
4-methoxy-1-benzofuran-6-ol	182.0811	0.7787	Positive	/	/	4.76	1.42	/	/
Scyphostatin A	512.3348	9.2759	Positive	/	/	4.68	1.48	/	/
p-Cresol sulfate	187.0062	4.5210	Negative	/	/	4.37	0.31	/	/
L-Carnitine	162.1123	0.7510	Positive	/	/	4.16	0.75	/	/
2-Hydroxy-3-methoxyestrone	424.3418	9.6654	Positive	/	/	/	/	4.68	1.36
LysoPC(P-18:0)^b^	552.3678	10.7062	Negative	/	/	/	/	4.48	1.22
Diosgenin	397.3101	9.9280	Positive	/	/	/	/	3.81	0.56
21-Deoxycortisol	347.2216	7.2521	Positive	/	/	/	/	3.60	1.19
12-Hydroxy-12-octadecanoylcarnitine	426.3573	10.1025	Positive	/	/	/	/	3.44	1.40
3alpha,12beta-Dihydroxy-11-oxo-5beta-cholan-24-oic Acid	424.3056	8.1553	Positive	/	/	/	/	3.19	1.74
Americine	584.2627	13.9012	Positive	/	/	/	/	2.97	1.89
LysoPE(20:4(8Z,11Z,14Z,17Z)/0:0) ^b^	502.2926	9.7375	Positive	/	/	/	/	2.92	1.23

^“a”^The ranking of top 20 is in descending order of VIP value. ^“b”^Means that the metabolites were correlated to hemolysis

As it were shown in Table 3 and Fig. 4. Compared with the NC group, OPD and OPD' group had similar results in terms of the type, quantity and VIP value of the differential metabolites. In top 20 differential metabolites, LysoPC account for 45% in OPD and OPD' group, while the phospholipid differential metabolites account for 75%. The abnormal increase of LysoPC and the disorder of phospholipid metabolism were consistent with the phenomena observed in in vivo hemolysis experiments.

### Lipidomic changes induced by OPD and OPD' in vivo

**Fig. 5:**
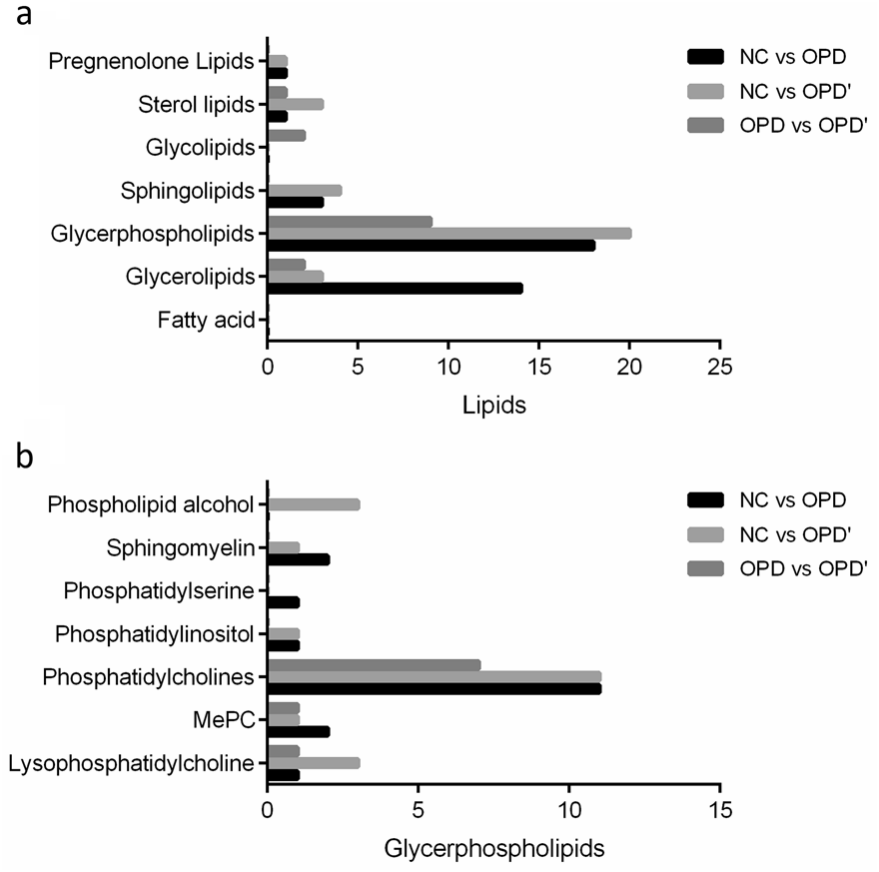
The distribution of different types of differential lipids in each comparison group. **a** Based on the differential lipids. **b** Based on the glycerphospholipids

A total of 82 lipids were identified as differential lipids. In each comparison group, the number of differential metabolites we found were 37 (NC vs OPD), 31 (NC vs OPD') and 14 (OPD vs OPD'), respectively. As were shown in Table 4 and Fig. 5. Glycerophospholipids (GPs) and glycerolipids (GLs) was significantly perturbed. Among GPs, phosphatidylcholines (PC) changes accounted for the largest proportion, which was 48.65% (NC vs OPD), 54.05% (NC vs OPD'), and 64.29% (OPD vs OPD') respectively. This was consistent with the changing trend of different metabolites in metabolomics. Among GLs, compared with the NC group, when treated with OPD, there is a significant difference in the change of GLs compared with OPD'. However, when OPD compared with OPD', changes of GLs was similar. In the three comparison groups, there was another pattern that makes people confused. Lysophosphatidylcholine (LPC), the main component that causes hemolysis, has been down-regulated when treated with OPD and OPD', this was not consistent with the observed hemolytic behavior.

**Table 4:** Detailed information of perturbed glycerophospholipids among different comparison group

Metabolites	m/z	Retention time (min)	Detection mode	(NC vs. OPD)		(NC vs. OPD')		(OPD vs. OPD')	
				VIP	FC	VIP	FC	VIP	FC
LPC(16:1)	538.3150	2.1049	Negative	1.80	0.54	1.69	0.68	/	/
MePC(19:4e)	566.3217	2.3768	Positive	1.94	1.50	/	/	1.51	0.82
MePC(38:8e)	824.5565	9.3435	Positive	1.32	1.72	1.23	1.45	/	/
PC(16:1_18:2)	800.5447	6.9216	Negative	1.46	0.57	/	/	/	/
PC(18:1_22:6)	876.5760	7.5938	Negative	1.42	0.58	1.38	0.67	/	/
PC(16:0_16:1)	776.5447	8.1717	Negative	1.05	0.63	/	/	1.54	1.76
PC(40:6)	834.6007	8.3262	Positive	1.13	0.71	/	/	/	/
PC(18:1_20:4)	852.5760	8.1460	Negative	2.86	0.75	/	/	2.60	1.31
PC(36:2)	786.6007	9.3590	Positive	8.17	1.24	6.62	1.12	/	/
PC(18:1e_16:0)	790.5967	9.6227	Negative	1.08	1.27	/	/	/	/
PC(18:1_18:1)	808.5827	9.3608	Positive	2.70	1.35	/	/	/	/
PC(18:0_20:5)	808.5851	9.3512	Positive	2.70	1.35	/	/	/	/
PC(34:1e)	746.6058	9.5947	Positive	1.07	1.39	1.11	1.28	/	/
PC(34:2e)	744.5902	9.0343	Positive	1.03	2.37	/	/	1.07	0.47
PI(19:0e)	613.3358	2.2930	Negative	1.02	0.79	1.54	0.73	/	/
PS(37:2)	800.5447	6.9216	Negative	1.46	0.57	/	/	/	/
SPH(d20:0)	330.3367	2.9837	Positive	1.50	1.56	/	/	/	/
SPH(t16:0)	290.2690	1.2942	Positive	1.52	3.07	1.23	2.16	/	/
cPA(18:2)	415.2255	2.5302	Negative	/	/	1.08	0.40	/	/
cPA(16:0)	391.2255	2.9310	Negative	/	/	1.41	0.53	/	/
cPA(18:0)	419.2568	3.4579	Negative	/	/	1.16	0.57	/	/
dMePE(40:7)	816.5549	7.8982	Negative	/	/	2.49	1.83	/	/
LPC(17:0)	554.3463	3.0918	Negative	/	/	1.57	0.69	/	/
LPC(18:2)	564.3307	2.4355	Negative	/	/	6.31	0.85	/	/
PC(20:4_20:4)	874.5604	6.4729	Negative	/	/	1.26	0.77	/	/
PC(18:0_18:2)	830.5917	9.3742	Negative	/	/	6.21	1.12	/	/
PC(16:0_18:1)	804.5760	9.2239	Negative	/	/	4.46	1.15	5.42	1.28
PC(34:0)	762.6007	9.8127	Positive	/	/	1.22	1.16	/	/
PC(18:1e_20:4)	838.5967	8.9016	Negative	/	/	1.26	1.25	/	/
PC(18:1e_16:0)	790.5967	9.6227	Negative	/	/	1.32	1.26	/	/
PC(16:1e_20:4)	810.5654	8.6947	Negative	/	/	1.13	1.79	/	/
LPC(18:0)	568.3620	3.3742	Negative	/	/	/	/	3.49	0.84
PC(34:1)	760.5851	9.2172	Positive	/	/	/	/	4.81	1.19
PC(16:0_16:0)	778.5604	9.2035	Negative	/	/	/	/	2.45	1.29
PC(14:0_18:2)	774.5291	6.7773	Negative	/	/	/	/	1.07	2.03

### Metabolic and Lipidomic pathway analysis

**Fig. 6:**
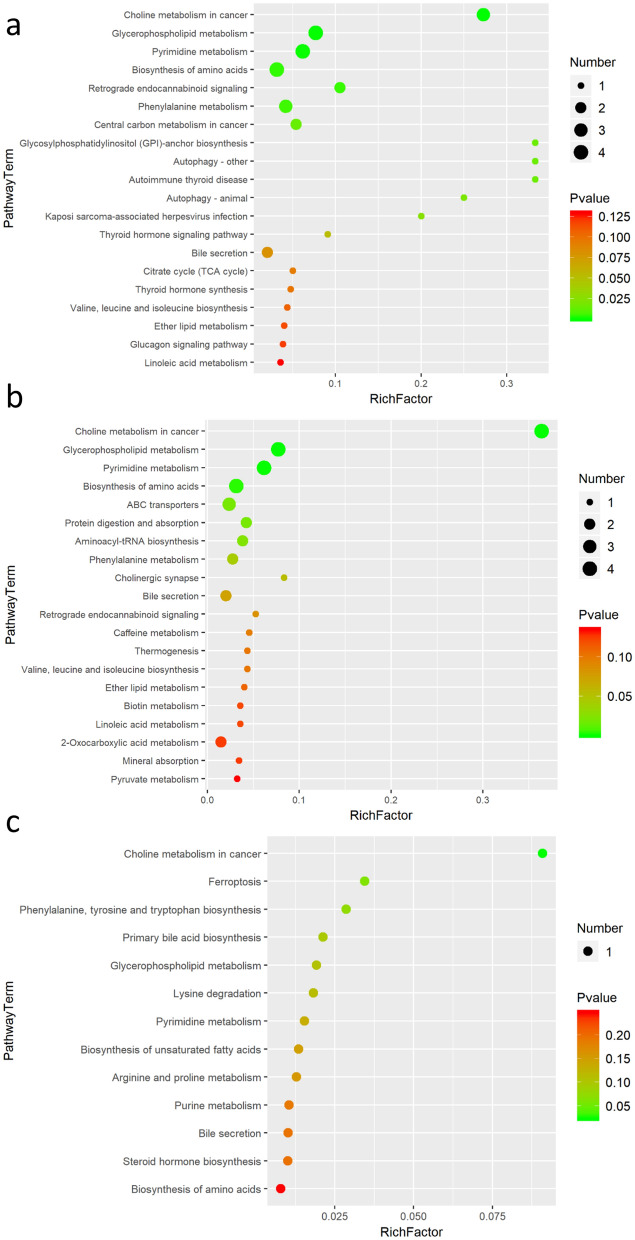
Top-20 Disturbed metabolic pathways. **a** NC vs OPD group. **b** NC vs OPD' group. **c** OPD vs OPD' group

In order to further explore the hemolysis mechanism of OPD and OPD', the different metabolites in the plasma of rats in the OPD and OPD' group were substituted into the progqenesis QI data processing platform for metabolic path analysis, as shown in Fig. 6. The results showed that the top-20 metabolic pathways disturbed by OPD or OPD' were similar. Since the pathway of lipid metabolites is insufficient in the KEGG website, not all differential metabolites can be reflected in KEGG pathway among the screened differential metabolites, this is also a challenge for current pathway analysis. However, the trend of the Fig. 6 is consistent with the results of the differential metabolites which were shown in Table 3, its predictions were still very informative.

Compared with NC group, choline metabolism in cancer and glycerophospholipid metabolism pathways were the most significantly enriched and largest number of differential metabolites gathered pathways in OPD and OPD' group, from a super class perspective, these two metabolic pathways were related to lipids and lipid-like molecules. As we all know, the main body of the cell membrane is the phospholipid bilayer and the fundamental cause of hemolysis of red blood cells is that the membrane was damaged. Therefore, we can speculate that the hemolysis caused by OPD and OPD' in vivo was related to the interference of glycerophospholipid metabolism. In addition, the pathways that were significantly affected also include pyrimidine metabolism, biosynthesis of amino acids, phenylalanine metabolism and bile secretion, these pathways not only have a certain relationship with the metabolism of glycerophospholipids, but may also participate in the metabolism and activation of OPD in vivo, which may result in the occurrence of OPD hemolysis.

## Discussion

From the perspective of the chemical structure and physical and chemical properties of OPD and OPD', hemolysis caused by OPD' can be reasonably explained by its chemical structure, but OPD cannot [24–26]. In another article we published [27], relationship of hemolytic behavior in vitro of OPD and OPD' was studied. The results showed that OPD' has hemolytic effect in vitro, but OPD does not. In other words, OPD' hemolysis occurred both in vivo and in vitro, but the OPD only occurred in vivo*.* This indicates that the structure of OPD' is more likely to cause hemolysis. Meanwhile, there is another possible explanation for this phenomenon, the one is that OPD is bio-transformed into OPD' or its analogues in vivo, the other one is that both OPD and OPD' were metabolized into more activated forms for hemolysis. The inconsistency of OPD’s hemolytic behavior in vivo and in vitro may be closely related to the metabolic process of OPD after administration.

When OPD was compared with OPD', the same differential metabolites based on comparison with NC group are neutralized, therefore, more details between OPD and OPD' in vivo metabolic processes were discovered. In its top 20 differential metabolites, LysoPC account for 20%, while cholic acid ranked first place (VIP value was 28.13, Fold change value was 1.72). In another article we submitted, molecular modeling was adopted and screen out a protein called Q9NPD5 which can interact with both OPD and OPD'. This protein belongs to organic anion transporters (OATP) family and it was closely related to cholic acid metabolism [28], existing studies have confirmed that the down-regulation of OATP1A2 and OATP1B3 in particular leads to abnormal fetal bile acid metabolism between maternal and fetal fetuses [29]. This may explain from another perspective how OPD and OPD' induce hemolysis. OATP1B3, which is highly expressed in the liver, is localized at the plasma membrane at the subcellular level. When OPD or OPD' enters the liver for metabolism, it binds to OATP1B3 [30], which disturbs the fluidity of the cell membrane and further induces hemolysis.

Published literature [31] shows that OPD can regulate the metabolism of glucose and lipids and improve the metabolic syndrome by interfering with the types and proportions of gut microbiota when taken orally. However, oral administration is very different from intravenous administration. Judging from the changes in the metabolic and lipid profile of different groups, the disorder of phospholipid metabolism dominates the causes of OPD and OPD' induced hemolysis [32, 33]. Statistics of all differential metabolites from two levels of super class and class were used to analyze the proportion of lipids and glycerophospholipids in the metabolic profile. In the NC vs OPD group, the ratio of super class level, i.e. lipids and lipid-like molecules, to all differential metabolites was 67/145, the ratio of class level, i.e. glycerophospholipids, to super class level’s metabolites was 42/67. In the other two groups, these two ratios were 60/124 (to all differential metabolites), 39/60 (to super class level’s metabolites) in NC vs OPD' group and 52/84 (to all differential metabolites), 10/52 (to super class level’s metabolites) in OPD vs OPD' group. Further analysis of sub-class metabolites at the class level, we found that at the sub-class level, the proportion of LysoPC was 18/42, 13/39 and 7/10 respectively. These ratios have significant statistical differences in comparison with other categories of differential metabolites in their corresponding levels. The metabolism of glycerophospholipid to lysophospholipid requires two steps of catalysis by phospholipase A2 and phospholipase B2, and the resulting lysophospholipid contains a hydrophobic hydrocarbon chain and a polar phosphate group. It was a very strong surfactant and has strong ability to destroy cell membranes [34]. But how OPD and OPD' participated in this process remains to be further explored.

Our further analysis of differential metabolites shows that it is very difficult to clearly point out a specific metabolite or pathway. The results of metabolomics and lipidomics experiments show that the changes of differential metabolites have obvious enrichment characteristics, and they are all enriched in the metabolism of phospholipids, including LysoPC, PC and cholic acid et.al., the relationship between these differential metabolites and hemolysis is very close. However, the lack of enrichment pathways related to these differential metabolites (lipids and lipid related metabolites) hinders the analysis of related pathways and the further work.

## Conclusion

UPLC-QE/MS was successfully applied to investigate the significant changes in plasma between OPD- and OPD'-treated rats. Both OPD and OPD' groups experienced hemolysis, subsequently, mechanism of differences in endogenous metabolites and lipids were investigated. Changes in endogenous differential metabolites and differential lipids, enrichment of differential metabolic pathways, and correlation analysis of differential metabolites and lipids all indicated that the causes of hemolysis by OPD and OPD' were closely related to the interference of phospholipid metabolism. This study provided a comprehensive description of metabolome and lipidomic changes between OPD- and OPD'-treated rats. However, the precise mechanism requires further exploration.

## Supplementary Information

**Additional file 1: Figure S1.** Volcano graph for screening differential metabolites. **Figure S2.** Volcano graph for screening differential lipids.

## Data Availability

All data used to support the findings of this study are available from the corresponding author upon request.

## Abbreviations

OPD: Ophiopogonin D
OPD': Ophiopogonin D’
UPLC: Ultra-high performance liquid chromatography
PCA: Principal component analysis
OPLS-DA: Orthogonal partial least square-discriminant analysis
PLS-DA: Partial least square-discriminant analysis
VIP: Variable importance in the projection
SMI: Shenmai injection
TCM: Traditional Chinese medicine
ROS: Reactive oxygen species
HGB: Hemoglobin
RBC: Red blood cell
RET: Reticulocyte
LysoPC: Lysophospholipids
MS: Mass spectrometry
HESI: Heated electrospray ionization
URO: Urobilinogen
OATP: Organic anion transporters
GPs: Glycerophospholipids
GLs: Glycerolipids
LPC: Lysophosphatidylcholine
