# Supplementary material for: Global metabolomic and lipidomic analysis reveals the potential mechanisms of hemolysis effect of Ophiopogonin D and Ophiopogonin D' in vivo
Source: Chin Med. 2021 Jan 6;16:3. doi: 10.1186/s13020-020-00412-z (PMC7787624; doi:10.1186/s13020-020-00412-z)
Supplement: Supplementary file 1 — Additional file 1: Figure S1. Volcano graph for screening differential metabolites. Figure S2. Volcano graph for screening differential lipids. [file 13020_2020_412_MOESM1_ESM.docx]

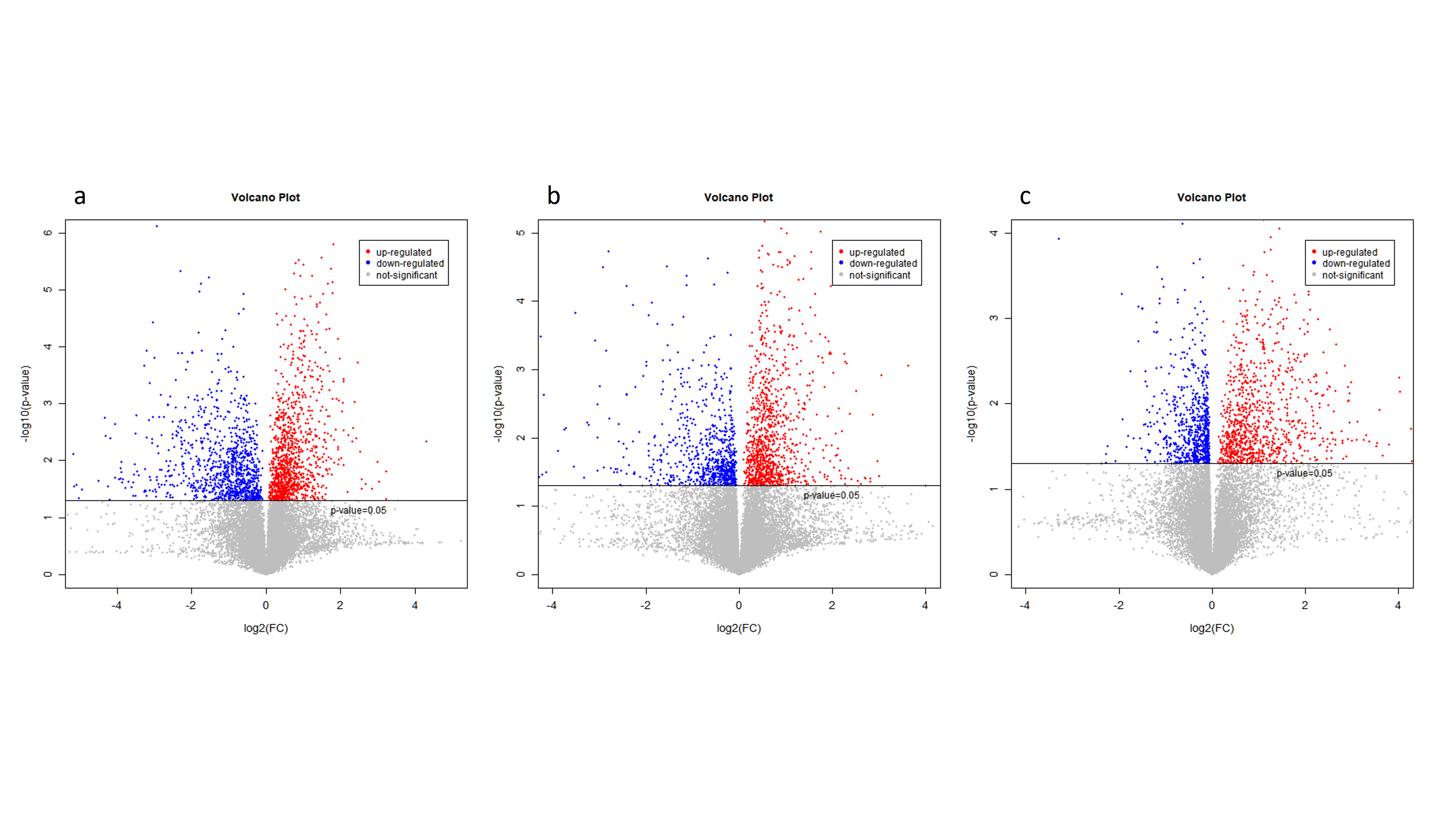


Figure. S1 Volcano graph for screening differential metabolites. (a) NC *vs* OPD. (b) NC *vs* OPD'. (c) OPD *vs* OPD'.


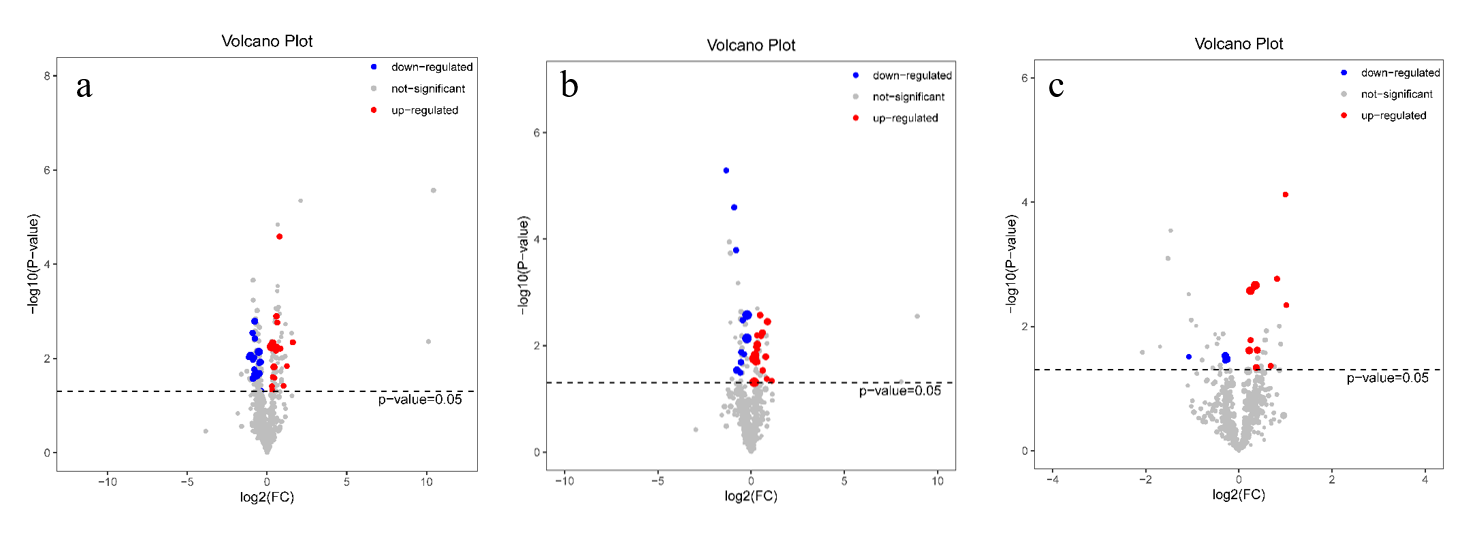


Figure. S2 Volcano graph for screening differential lipids. (a) NC *vs* OPD. (b) NC *vs* OPD'. (c) OPD *vs* OPD'.
